# Supplementary figures and images for: Extracellular space preservation aids the connectomic analysis of neural circuits
Source: eLife. 2015 Dec 9;4:e08206. doi: 10.7554/eLife.08206 (PMC4764589; doi:10.7554/eLife.08206)

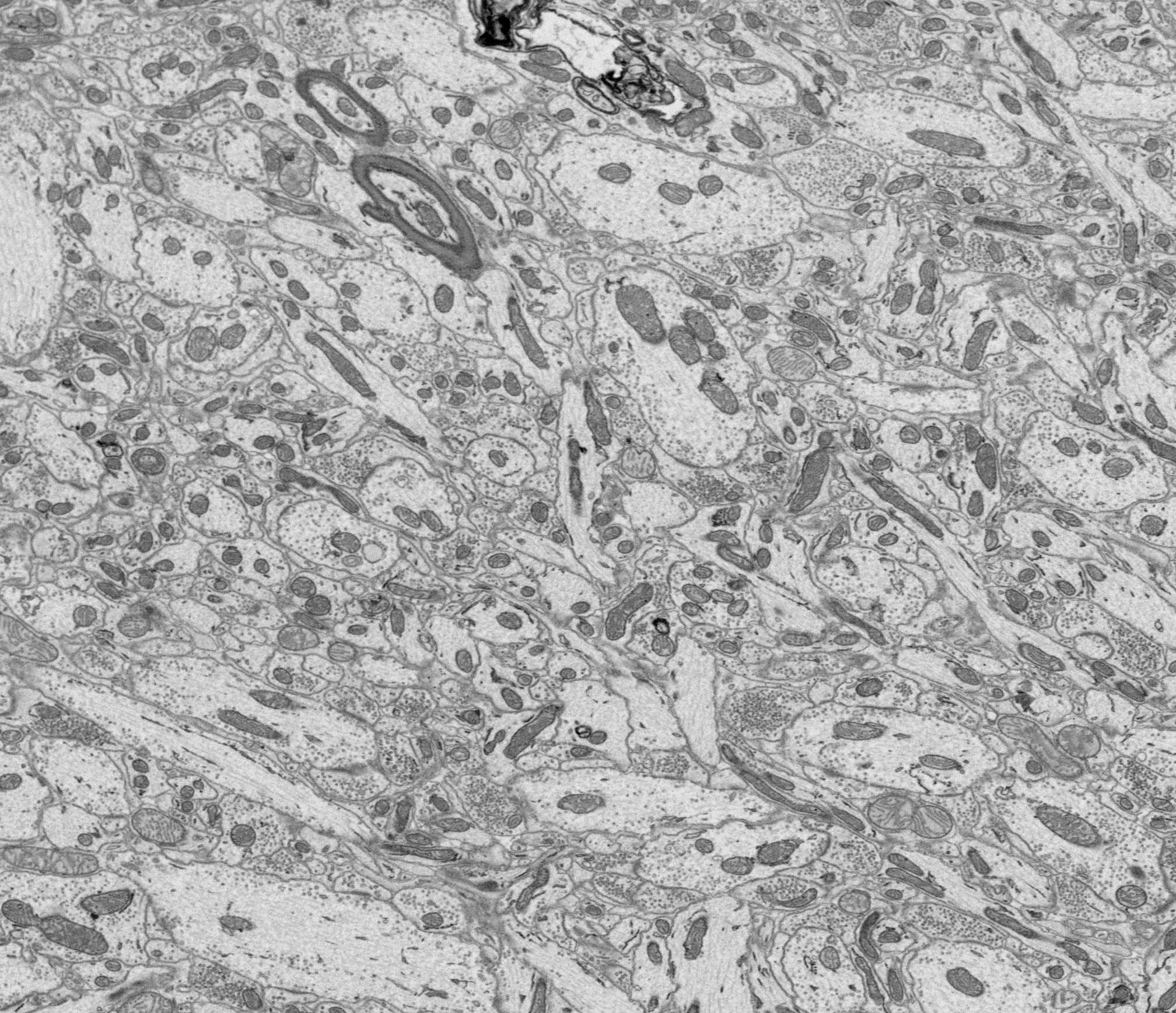

Supplement: Figure 2—source data 1. — TIFF stack viewable using ImageJ. ECS: Extracellular space. DOI: http://dx.doi.org/10.7554/eLife.08206.007 [file elife-08206-fig2-data1.tif]

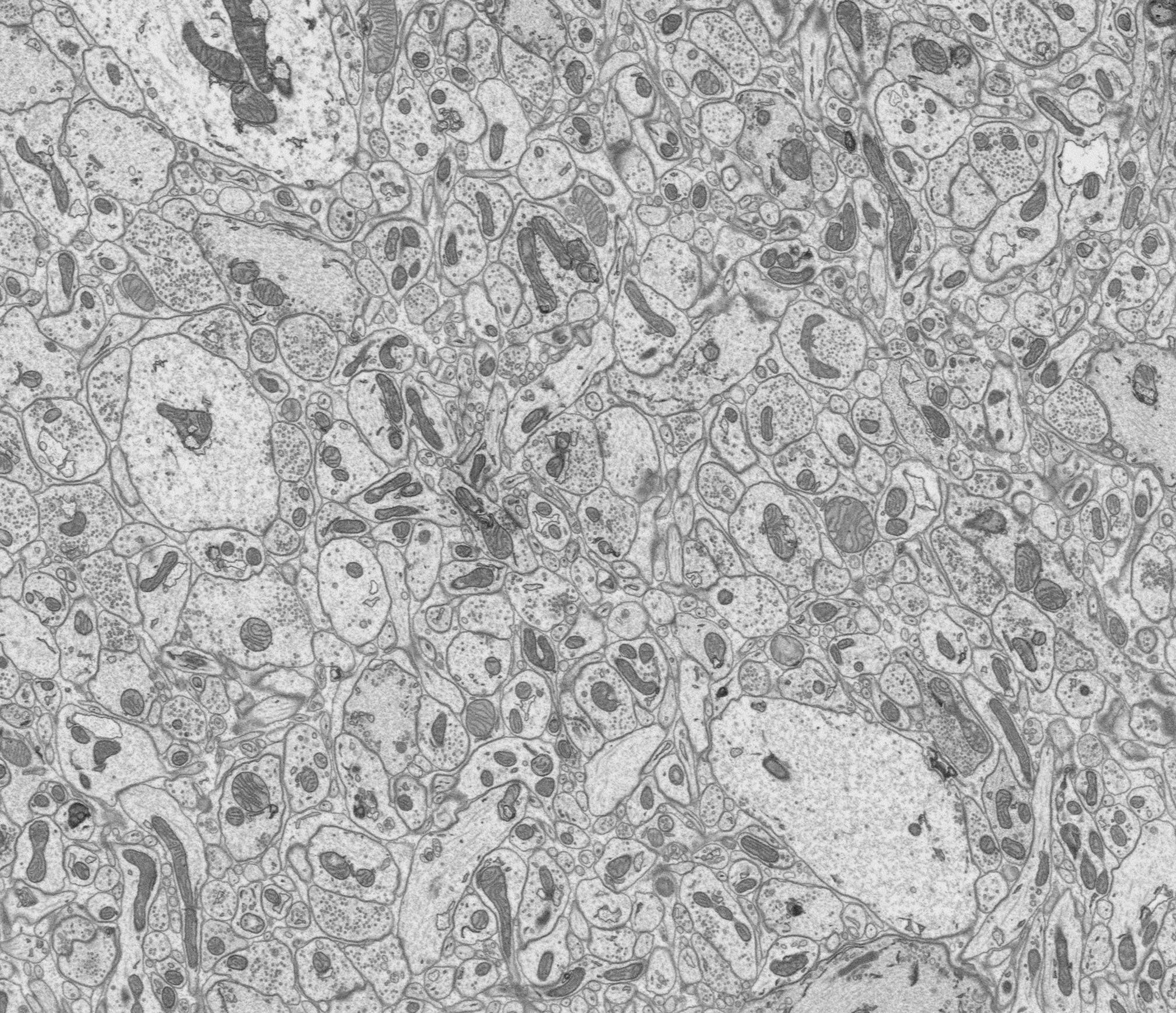

Supplement: Figure 2—source data 2. — TIFF stack viewable using ImageJ. ECS: Extracellular space. DOI: http://dx.doi.org/10.7554/eLife.08206.008 [file elife-08206-fig2-data2.tif]

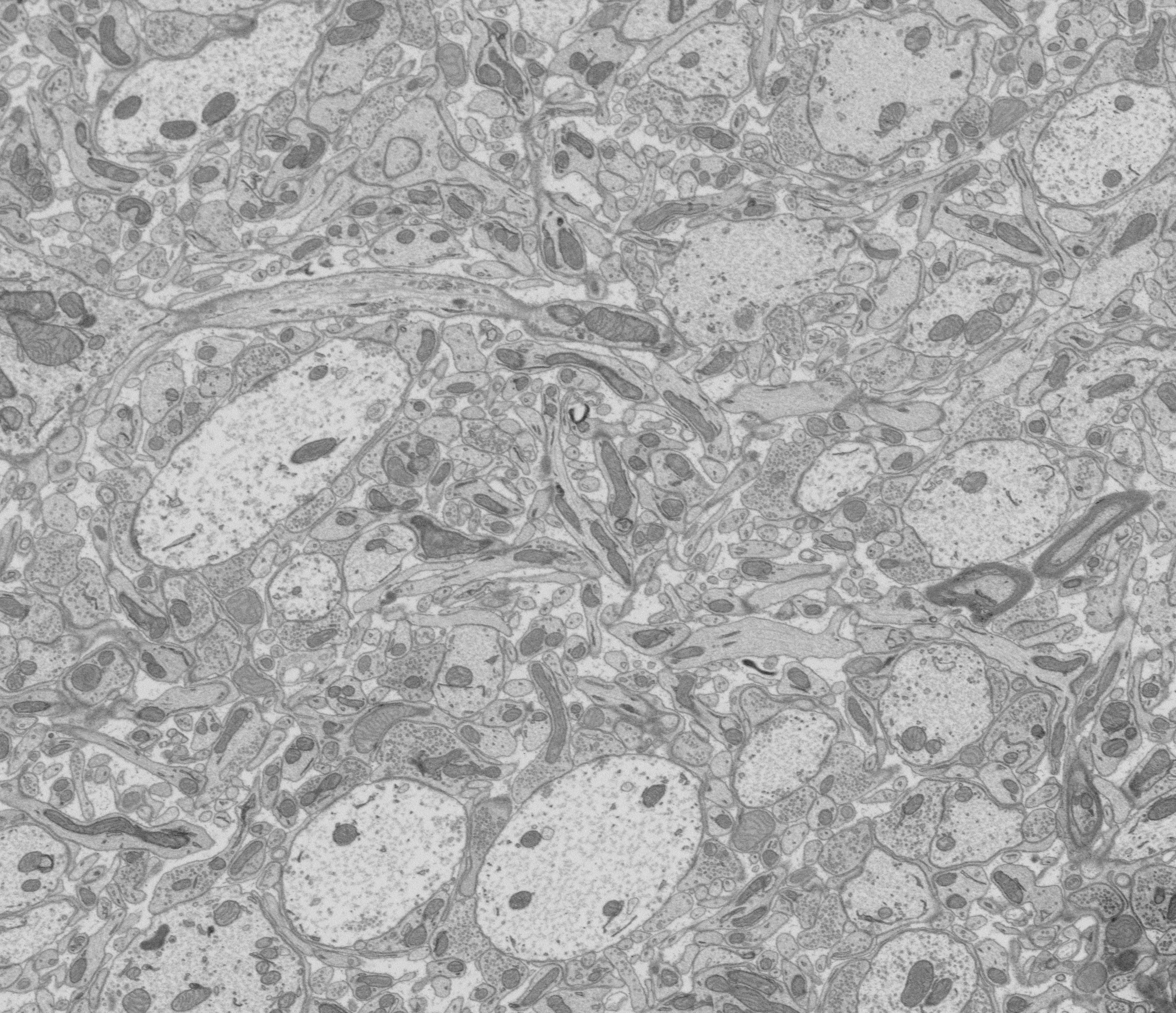

Supplement: Figure 2—source data 3. — TIFF stack viewable using ImageJ. ECS: Extracellular space. DOI: http://dx.doi.org/10.7554/eLife.08206.009 [file elife-08206-fig2-data3.tif]

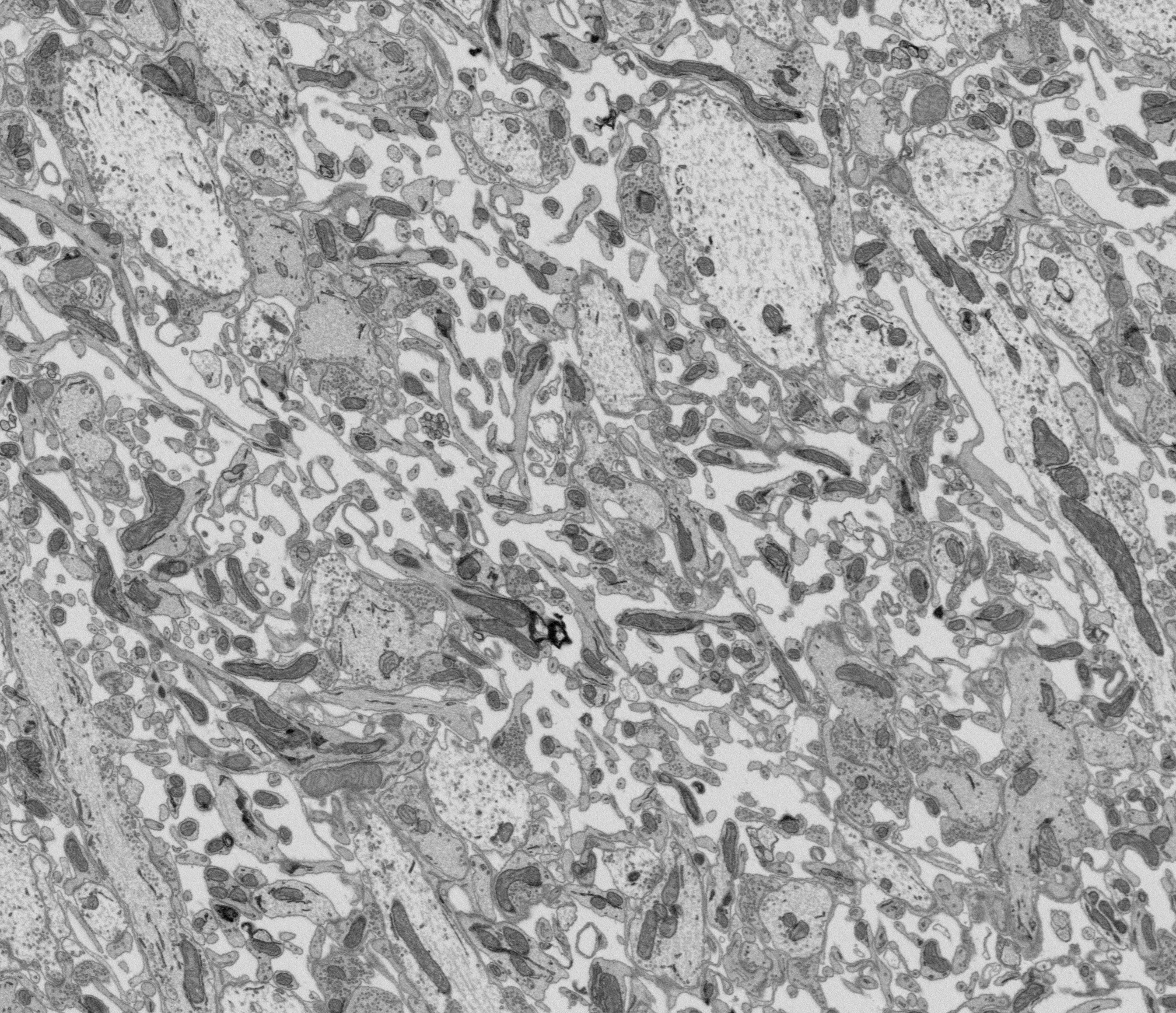

Supplement: Figure 2—source data 4. — TIFF stack viewable using ImageJ. ECS: Extracellular space. DOI: http://dx.doi.org/10.7554/eLife.08206.010 [file elife-08206-fig2-data4.tif]

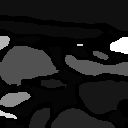

Supplement: Figure 3—source data 3. — DOI: http://dx.doi.org/10.7554/eLife.08206.015 [file elife-08206-fig3-data3.zip › M0007_33_groundtruth_cleaned_x0017_y0019_z0002.tif]

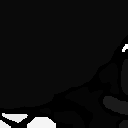

Supplement: Figure 3—source data 3. — DOI: http://dx.doi.org/10.7554/eLife.08206.015 [file elife-08206-fig3-data3.zip › M0007_33_groundtruth_cleaned_x0017_y0023_z0001.tif]

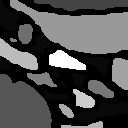

Supplement: Figure 3—source data 3. — DOI: http://dx.doi.org/10.7554/eLife.08206.015 [file elife-08206-fig3-data3.zip › M0007_33_groundtruth_cleaned_x0019_y0022_z0002.tif]

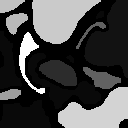

Supplement: Figure 3—source data 3. — DOI: http://dx.doi.org/10.7554/eLife.08206.015 [file elife-08206-fig3-data3.zip › M0007_33_groundtruth_cleaned_x0022_y0018_z0001.tif]

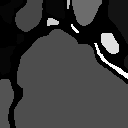

Supplement: Figure 3—source data 3. — DOI: http://dx.doi.org/10.7554/eLife.08206.015 [file elife-08206-fig3-data3.zip › M0007_33_groundtruth_cleaned_x0022_y0023_z0001.tif]

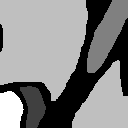

Supplement: Figure 3—source data 3. — DOI: http://dx.doi.org/10.7554/eLife.08206.015 [file elife-08206-fig3-data3.zip › M0007_33_groundtruth_cleaned_x0022_y0023_z0002.tif]

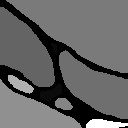

Supplement: Figure 3—source data 4. — DOI: http://dx.doi.org/10.7554/eLife.08206.016 [file elife-08206-fig3-data4.zip › M0027_11_groundtruth_cleaned_x0013_y0015_z0003.tif]

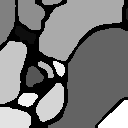

Supplement: Figure 3—source data 4. — DOI: http://dx.doi.org/10.7554/eLife.08206.016 [file elife-08206-fig3-data4.zip › M0027_11_groundtruth_cleaned_x0013_y0020_z0003.tif]

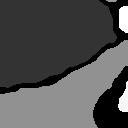

Supplement: Figure 3—source data 4. — DOI: http://dx.doi.org/10.7554/eLife.08206.016 [file elife-08206-fig3-data4.zip › M0027_11_groundtruth_cleaned_x0016_y0017_z0004.tif]

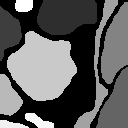

Supplement: Figure 3—source data 4. — DOI: http://dx.doi.org/10.7554/eLife.08206.016 [file elife-08206-fig3-data4.zip › M0027_11_groundtruth_cleaned_x0018_y0015_z0003.tif]

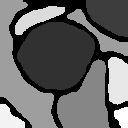

Supplement: Figure 3—source data 4. — DOI: http://dx.doi.org/10.7554/eLife.08206.016 [file elife-08206-fig3-data4.zip › M0027_11_groundtruth_cleaned_x0018_y0020_z0003.tif]

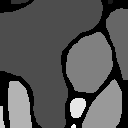

Supplement: Figure 3—source data 4. — DOI: http://dx.doi.org/10.7554/eLife.08206.016 [file elife-08206-fig3-data4.zip › M0027_11_groundtruth_cleaned_x0018_y0020_z0004.tif]

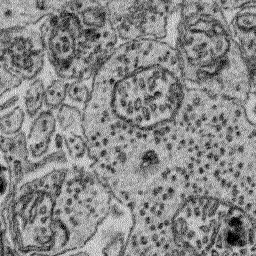

Supplement: Figure 4—source data 1. — TIFF stack viewable using ImageJ. Slice #128 in the stack indicates the location of the tight contact. DOI: http://dx.doi.org/10.7554/eLife.08206.019 [file elife-08206-fig4-data1.tif]

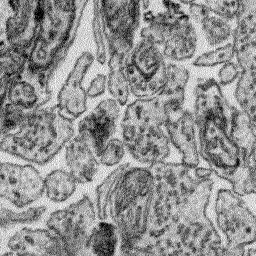

Supplement: Figure 4—source data 2. — TIFF stack viewable using ImageJ. Slice #128 in the stack indicates the location of the tight contact. DOI: http://dx.doi.org/10.7554/eLife.08206.020 [file elife-08206-fig4-data2.tif]

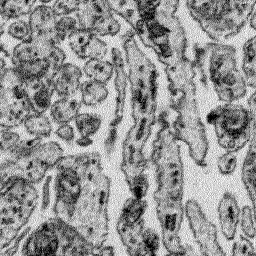

Supplement: Figure 4—source data 3. — TIFF stack viewable using ImageJ. Slice #128 in the stack indicates the location of the cleft contact. DOI: http://dx.doi.org/10.7554/eLife.08206.021 [file elife-08206-fig4-data3.tif]
